# Life after pelvic organ prolapse surgery: a qualitative study in Amhara region, Ethiopia

**DOI:** 10.1186/s12905-018-0568-2

**Published:** 2018-05-29

**Authors:** Janne L. Gjerde, Guri Rortveit, Mulat Adefris, Tadesse Belayneh, Astrid Blystad

**Affiliations:** 1 Research group for Global Health Anthropology, Centre for International Health, Department of Global Public Health and Primary Care, University of Bergen, Bergen, Norway; 2 Department of Obstetrics and Gynecology, Haukeland University Hospital, Bergen, Norway; 3 Research Group for General Practice, Department of Global Public Health and Primary Care, University of Bergen, Bergen, Norway; 4 Research Group for General Practice, Uni Research Health, Bergen, Norway; 5 Department of Obstetrics and Gynecology, College of Medicine and Health Sciences, University of Gondar, Gondar, Ethiopia; 6 Department of Anaesthesiology, College of Medicine and Health Sciences, University of Gondar, Gondar, Ethiopia; 7 Department of Global Public Health and Primary Care, University of Bergen, PO Box 7804, 5020 Bergen, Norway

**Keywords:** Ethiopia, Experience, Pelvic organ prolapse, Recovery, Reintegration, Surgery

## Abstract

**Background:**

Women living in resource constrained settings often have limited knowledge of and access to surgical treatment for pelvic organ prolapse. Additionally, little is known about experiences during recovery periods or about the reintegration process for women who do gain access to medical services, including surgery. This study aimed to explore women’s experiences related to recovery and reintegration after free surgical treatment for pelvic organ prolapse in a resource-constrained setting.

**Methods:**

The study had a qualitative design and used in-depth interviews in the data collection with a purposive sample of 25 participants, including 12 women with pelvic organ prolapse. Recruitment took place at the University of Gondar Hospital, Ethiopia, where women with pelvic organ prolapse had been admitted for free surgical treatment. In-depth interviews were carried out with women at the hospital prior to surgery and in their homes 5–9 months following surgery. Interviews were also conducted with health-care providers (8), representatives from relevant organizations (3), and health authorities (2). The fieldwork was carried out in close collaboration with a local female interpreter.

**Results:**

The majority of the women experienced a transformation after prolapse surgery. They went from a life dominated by fear of disclosure, discrimination, and divorce due to what was perceived as a shameful and strongly prohibitive condition both physically and socially, to a life of gradually regained physical health and reintegration into a social life. The strong mobilization of family-networks for most of the women facilitated work-related help and social support during the immediate post-surgery period as well as on a long-term basis. The women with less extensive social networks expressed greater challenges, and some struggled to meet their basic needs. All the women openly disclosed their health condition after surgery, and several actively engaged in creating awareness about the condition.

**Conclusions:**

Free surgical treatment substantially improved the health and social life for most of the study participants. The impact of the surgery extended to the communities in which the women lived through increased openness and awareness and thus had the potential to ensure increased disclosure among other women who suffer from this treatable condition.

**Electronic supplementary material:**

The online version of this article (10.1186/s12905-018-0568-2) contains supplementary material, which is available to authorized users.

## Background

Symptomatic pelvic organ prolapse (hereafter ‘prolapse’) occurs in 6–7% of women in the United States, [1, 2] with a 12% lifetime risk of undergoing surgical treatment [3]. Pregnancy and childbirth are important risk factors for prolapse, [2, 4] and the risk increases with the number of vaginal deliveries [5]. Prolapse may be more common and may more severely affect daily living in resource-constrained settings due to high fertility rates, early-age deliveries, limited access to obstetric care, and the rigors of manual work [6, 7]; however, information from such settings on prevalence and risk factors remains limited. The few studies on prolapse available from low- and middle-income countries have reported prevalence rates ranging from 3 to 56% and include research on prolapse symptoms and prolapse verified by pelvic examination [7–9]. In Ethiopia, a population-based pilot study conducted in the same research area as this study reported a 55% prevalence of stages II–IV prolapse among participants who had undergone pelvic examinations using the simplified Pelvic Organ Prolapse Quantification (POPQ) staging system [6, 10]. Other studies from Ethiopia have suggested an association between prolapse stage and age, and parity and occupation [11] as well as between prolapse and underweight conditions, lack of formal education, and childbirth without health professionals [12]..

Independent of place of residence, prolapse has been reported to negatively affect the quality of life including sexual function and body image [13–15]. Findings from rural parts of Ethiopia and other resource-constrained settings suggest the severity of the social consequences of prolapse, including the possibility of divorce and discrimination [16–20]. Like urinary incontinence and obstetric fistula, prolapse is commonly considered by rural Ethiopian women as a sensitive topic as well as a shameful and repulsive condition [16, 21, 22]. Recent publications from the United States and Australia offer strong evidence of quality of life improvements following vaginal surgery for prolapse, including improved sexual function and body image [23–26] .However, there is an ongoing and comprehensive debate among medical experts worldwide as to which type of surgical method gives the most desirable long-term outcome [26, 27]..

Little is known about how women in resource-constrained settings experience the recovery period following prolapse surgery. Moreover, there is limited knowledge about women’s processes of reintegration into their communities after the surgery. Studies on obstetric fistula in Ethiopia and Kenya suggest that surgical repair only represents the starting point for affected women who wish to seek social reintegration [22, 28] .The current study aimed to explore women’s experiences related to recovery and reintegration following the provision of free surgical treatment for prolapse in a rural Ethiopian setting.

## Methods

### Study setting

The current study took place in the Amhara region of north-western Ethiopia. Roughly 20% of births among rural women in Ethiopia are attended by skilled personnel or occur at health facilities. The nation’s fertility rate is 4.6 children per woman, and the maternal-mortality ratio is 412 deaths per 100,000 live births [29]. Rural Ethiopian health facilities are in general poorly equipped and lack adequate emergency obstetric services [30]. The Amhara people, who primarily practice Orthodox Christianity and speak Amharic as their first language, are the majority ethnic group in the region [31]. The median female age upon first marriage in the Amhara region is 16.2 years, and around 55% of the women are illiterate [29]..

The present study, which was conducted in 2015–16, featured repeated visits to the field. The three-months-long first part of the fieldwork was conducted at the University of Gondar Hospital (henceforth ‘the hospital’), a referral teaching hospital located in the city of Gondar. The second part of the study, which lasted 4 months, took place in semi-urban and rural parts of the districts of Dabat and Debark, located 78 and 106 km north of Gondar, respectively. Free surgical treatment was introduced at the hospital at the time of the fieldwork and was offered to women with prolapse. It was initiated and funded by the hospital and UNFPA in collaboration with two non-governmental organizations (NGOs). The women were informed about prolapse and the possibility of free treatment from health-extension workers (HEWs) in the communities. Those who were found eligible for surgical treatment were selected at the district level and sent to the hospital in small groups.

### Participant recruitment and data collection

The study had a qualitative, explorative approach and included 25 participants (Table 1). The first part of the fieldwork was conducted at the hospital where women with prolapse were admitted for surgery. Women who had undergone prolapse surgery were interviewed and recruited for follow-up visits in their homes after expected recovery. The criteria for follow-up included prolapse surgery and the accessibility of the women’s homes. The first author carried out participant observations at the hospital, which were primarily conducted in connection with another sub-study focusing on health-seeking behaviours in the same patient group. The author’s presence at the ward also facilitated the recruitment of informants for the present sub-study and secured access to the informants’ medical histories. Health-care providers and a representative from one of the organizations involved in the newly introduced free prolapse-treatment initiative were also interviewed at the hospital.

**Table 1:** Study participants according to recruitment place

Hospital	Number	Community	Number
Women admitted for surgical treatment of prolapse ^a^	8	Follow-up of women having undergone surgical treatment at hospital	8
Health care providers	2	Women who had undergone surgical treatment at the same facility and time period	4
Representative from organization affiliated with the hospital	1	Health extension workers working at community level	4
		Health care providers working at health centre level	2
		Representatives from international NGO	2
		Representatives from the health authorities at district level	2
Total interviews	11		22

^a^These women were primarily taking part in a sub-study that focused on the experience of living with prolapse [16], and were recruited for a follow-up visit in their homes

The second part of the study took place in the women’s communities and included home visits 5–9 months after their surgeries. HEWs who were involved in community mobilization activities in connection with prolapse surgery were interviewed. They were engaged in the identification of potential prolapse cases and referred women with suspected prolapse to the district level. Health-care workers at the health-centre level, as well as representatives from an international NGO and representatives from the health authorities at the district level, all of whom were involved in the newly introduced free prolapse-treatment initiative, were also interviewed to provide contextual information for the study.

All interviews were performed in close collaboration with a local female interpreter who was familiar with the language, culture, and respectful conduct in the area. The interviews were conducted in Amharic with continuous translation from English to Amharic and vice versa between the researcher and the informants. Semi-structured interview guides with open-ended questions were used (see Additional file 1). The interviews, which were held either inside or outside the women’s homes, lasted from 1 to 2 h with the aim of allowing the informants to speak freely and with few interruptions. All the interviews at the hospital were held in a private room on the ward while the interviews with the health-care providers and stakeholders in the communities were held in a private room at their work facilities.

### Analysis

The analysis took place throughout the data-collection process and during a rigorous analytical phase that followed the completion of the fieldwork. All interviews were audio-recorded, transcribed verbatim to Amharic and translated into English. The completed material was carefully reviewed to identify core themes [32]. The subsequent post-fieldwork analysis concretized the initially identified themes into categories of meaningful units followed by coding of the material line-by-line [33]. Each sub-category identified during the first phase was scrutinized for central patterns and ‘case-stories’ as well as for potential nuances, and ambivalence and contradictions. The full data set was then imported into NVivo 11, a qualitative data-analysis software tool that was employed to organize the material.

### Ethical considerations

Ethical approvals were obtained from the Regional Ethics Review Board in Norway and the University of Gondar in Ethiopia. With the assistance of the interpreter, all patients on the ward were provided with information about the study, the role of the first author’s participant observation, and their rights not to participate or be observed. The aim and purpose of the study, as well as the contents of the consent form, were explained to the research participants prior to all interviews. Written or oral consent to participate was obtained, depending on literacy status, and the utmost care was taken to secure privacy and confidentiality during the research process. Two patients at the hospital declined to participate, and two women were lost to follow-up with the research team due to distance or lack of accessible roads to their homes.

## Results

The main category of informants consisted of 12 women (Table 2), all of whom had undergone surgical treatment for prolapse. Upon admission, 11 of the 12 women were diagnosed with stage III prolapse of the uterus, bladder and/or rectum according to the simplified POPQ staging system [10]. The majority had undergone a vaginal hysterectomy and sacrospinous fixation and/or an anterior or posterior colporrhaphy under spinal anaesthesia. None received oestrogen therapy prior to or after the surgery. All women, except for one, returned to the hospital for their follow-up appointments scheduled by the hospital staff to take place 1–3 months after surgery. Two women got confirmed vault prolapse during their hospital appointment. During our follow-up interviews 5–9 months after the surgery, half of the women explained that they had a highly improved health condition with few or no complaints, whereas the other half still had some challenges. Some again felt that something was emerging from their vagina and others could not control their urination.

**Table 2:** Characteristics of main participants

Mean age	43.3 years [range 32–60]
Marital status	
Married	8
Divorced	3
Widowed	1
Mean age at first marriage	13 years [range 7–19]
Education	
No school / illiterate	10
Literate	2
Occupation	
Housework	6
Housework and farming activities	5
Daily laborer	1
Mean number of children	3.6 children [range 0–8]
Mean age at first delivery	18.5 [range 13–24]
Place of delivery	
All deliveries at home	10
One or two deliveries at health facility	2

### Recovery

Although only half the women had fully recovered 5–9 months after the surgery, nearly all expressed appreciation for their present situation:

*I used to have to walk by holding the prolapse; I couldn’t walk like I wanted to. I had a lot of problems. But I’m thankful after the surgery; it was a big change. I had suffered for six years.* (32-year-old married woman)

Following surgery, the women were told to avoid heavy strains and lifting for their lifetime and to avoid sexual intercourse for up to 3 months. The women in this region of Ethiopia normally have full responsibility for household chores, including the procuring of water and firewood and occasionally taking part in farming activities. Collecting water for many of the women involved daily multi-hour walks carrying 20–30 l on their backs. The avoidance of chores after the surgery, hence, depended on receiving substantial support from their neighbours, family members, and more distant relatives. The customary support systems related to illness and births were mobilized in these cases; indeed, all the informants explained that they had received substantial support when they returned from the hospital:

*Until my [hospital follow-up] appointment was approaching, I lay on my bed while my neighbours fed my family and me and took turns doing my chores.* (52-year-old married woman).

After a month or more of rest, the external support system slowly decreased as relatives and neighbours returned to their own homes. The condition nonetheless required continuous support to avoid having the woman return to heavy chores. The assistance was commonly provided by immediate family members:

*I don’t work at home much anymore. My daughter is cleaning the house, inside and outside. I used to help with the farming, but now I’ve stopped doing that too. My husband manages by himself.* (40-year-old married woman).

Many of the women expressed fear of returning to their previous heavy chores:

*I feel I am cured now. But I don’t doubt that if I start doing heavy work again I would feel sick. Now I fear to do heavy work*. (32-year-old married woman).

Divorced and widowed women largely lived by themselves and depended on their children or on extended family members to decrease their work burdens after the initial healing period. These women who were socially vulnerable found it more challenging to cope, not the least in cases of delayed healing:

*I used to work as a labourer preparing food for people. I don’t feel good enough yet to start work. I can’t even walk long distances or lift anything, and I don’t have anyone to help me. I ask neighbourhood children to fetch water for me. I no longer have any money saved, and I feel that darkness has surrounded me. I had to send my two young children to work in other people’s houses. If I sit like this, how can I feed myself? I’m still waiting to recover, and then I’ll start working again.* (39-year-old divorced woman).

Although many of the women interviewed referred to stories of other women who had been unable to abstain from sex upon their return home to their husbands, the women interviewed in the present study seemed to have husbands who accepted their conditions:

*We haven’t had sex for five months now, and he hasn’t forced me. He hasn’t asked me during this time—he can see the pain I’ve been in*. (35-year-old married woman).

The HEW’s overall impression was that the women made strong attempts to follow the advice they were given by the hospital after the surgery, but they also mentioned cases where the woman had no choice but to work due to lack of available support.

### Disclosure

Most of the women had disclosed their conditions to very few confidantes prior to their surgery. Typically, they shared with their mother, sister or close friend in addition to the HEW. All the married women disclosed their conditions to their husbands, but the majority did so at a late date and only after the condition had worsened. Before disclosing, some had considered divorce to escape the shame they experienced. Although many of the husbands were said to be supportive, they commonly had a limited ability to assist their wives. The divorced women had chosen to leave their husbands because of the prolapse, either due to being ignored and disrespected or because they were unable to fulfil their expected roles as wives due to their condition.

The period when the informants were brought together with their fellow prolapse sufferers in connection with the hospitalization process proved to be vital for disclosure. During the time they spent together, they established close ties:

*Once we travelled together to the hospital, we didn’t talk about anything but our conditions for five days. We didn’t know each other, but we still talked a lot. We laughed and discussed like mothers and daughters do. We all shared our experiences with the prolapse.* (35-year-old married woman).

Once the women had returned from the hospital, many faced questions about the treatment they had received, and the majority decided to be open about what they had experienced:

*After the treatment, people asked me where I’d been and what had happened to me. I told them all about my condition and the treatment I’d received*. (39-year-old divorced woman).

The response from their neighbours and relatives was mainly positive; many expressed sympathy and wondered why they had kept the prolapse a secret for so long. All the women expressed great relief related to their newly gained experience of openness:

*Why should I feel shame now? I’ve seen the light. I hid the condition for 20 years. But now that I’ve had the treatment, I’ve escaped the pain. I feel relaxed now*. (40-year-old married woman).

The HEW’s also reported noticing a change in openness among women in the communities:

*Women have become more open to talk to us about their problems. We usually speak about prolapse when we get a chance in the Church or at community meetings. Then women come directly to us and say: “I have this problem. I kept it to myself.”* (HEW 2).

### Reintegration

Many of the women told of the extreme social restrictions caused by the prolapses, and explained how they had been unable to fulfil the social roles expected of them. When living with the prolapse condition, the crucial inviting and hosting of people for holidays had become increasingly challenging for many since food preparation often involved heavy lifting, including the procurement of extra water for the occasion. Walking longer distances to attend social gatherings, such as funerals or events with far-off relatives, had also become problematic. One woman, who had found it difficult to sit among people because of the pain and itching of her ulcerated prolapse, and from her frequent need to urinate, explained the transformation she had experienced:

*It used to be embarrassing to sit with people outside or inside others’ homes. It was shameful for me to eat, drink and [suddenly] go outside to urinate. I stopped attending social gatherings because of that. That situation cannot be compared to the present. I urinate less frequently, and I can sit how I want to sit and talk with relatives without a problem*. (35-year-old married woman).

Not all the women felt ready to fully engage in every social gathering at the time of the study.

*I haven't started visiting relatives yet because the doctor told me not to go anywhere far for six months. I might start having long journeys and visit relatives soon, God willing.* (45-year-old married woman).

Most, however, lived relatively close to other women they had been hospitalized with, and many of them stayed in touch:

*One of these women is my neighbour. Now we drink coffee and fetch water together. Both of us trust each other*. (40-year-old married woman) *We all have a wish to meet after the surgery, and we have planned to meet at every holiday.* (39-year-old divorced woman).

### Engagement

The increased openness among the women after their return home from the hospital had implications in their local communities. One woman explained that prolapse was now a common and unproblematic topic to discuss with friends. Indeed, this was reflected by the health-care workers:

*If the new initiative goes on, women won’t be hiding this condition anymore. It is a way to avoid women’s discrimination.* (Health-care provider, Health Clinic).

After returning from the hospital, several of the study participants were approached in secrecy by women in the community who asked for details about the condition and about the treatment they had received:

*A lot of women out there haven’t yet received treatment [for prolapse]; they hide their condition and pain. One woman from my village came to my home—she had been too ashamed to tell anybody about her condition. I asked her why she felt ashamed. We don’t have to hide this condition these days. After our conversation, she talked to the health-extension worker and was sent to the hospital for surgery. She later became a very dear friend*. (32-year-old married woman).

As a part of the new prolapse initiative, the selected women who had been treated were now trained as maternal-health advocates. In addition to spreading information about maternal-health matters in their communities, the advocates were trained to seek out and register women who suffered from prolapse:

*When mothers return home after being treated, we train them, and the health bureau also gives them a checklist. They mainly work alongside the community health workers (HEWs), and we encourage them to participate in different activities of the project. We also have a radio channel which facilitates the mobilization process.* (NGO representative).

The women who had yet to receive training were also eager to spread information about prolapse and to encourage others to get treated:

*When I go to fetch water, I inform women [about prolapse]. I also tell their husbands to take their wives to the hospital if they are sick. I explain how I got better. I advise them to seek medical care because the government supports us now. Many women hide their problems, and that’s bad for their well-being. I suffered a lot because I concealed my problem*. (40-year-old divorced woman).

## Discussion

### Main findings

The opportunity for treatment proved to have substantial implications for the rural Amhara women who were followed up in the present study. The majority received substantial practical support during the recovery period and experienced understanding after disclosing their condition. For many of the women, the disclosure and awareness related to prolapse became an important activity to ensure that other women who were suffering alone would learn about prolapse and the available free treatment. The increased disclosure led to a surprising degree of openness and awareness about the condition in the communities in which the study took place. Not all the women, however, experienced improvements in their lives, especially the ones who lived alone and had a problematic healing process. For these women, life continued to be a struggle.

### Interpretation of results

The key issues raised by the women in the study were that the surgery alleviated them of the most pressing concerns of discrimination, rejection, and divorce [16]. The sharing of experiences and the relationships that developed among the women who were recruited for treatment proved to be important for the women’s increasing sense of knowledge about the condition and sense of empowerment as well as the subsequent openness and disclosure of the condition. The current study thus indicates the immense transformation that can be facilitated when groups of women are recruited jointly for surgery and go through a joint learning process. These factors speak to the importance of the surgery beyond the physical repair itself; the regaining of a social life lies at the core of the stories these women tell. The emphasis of the social dimensions has similarities with experiences from other health initiatives, for example within the field of HIV/AIDS, where beyond the antiretroviral therapy, the training, employment, and empowerment of local mothers who live with HIV have led to reduced HIV-related stigma and discrimination [34]..

In a study of women who were treated for obstetric fistula in Kenya [28], finding a sense of belonging after their treatment and reintegration into the community following surgery depended on their available support mechanisms. Although many of the prolapse sufferers in the present study experienced extensive support after surgery, the available help still proved insufficient for some due to limited social networks. Among tuberculosis patients being treated in Ethiopia [35], the support from family and community members similarly proved crucial although many patients found that the level of support dropped during the course of treatment. Thus, it proved difficult for them to cope, especially the ones with limited human or material resources.

In line with scholars within critical medical anthropology [36–38], we argue that poverty and marginalization increase the likelihood of illness and suffering, such as when life conditions seen as insignificant hamper the potential for receiving adequate care for serious conditions of prolapse. Taking into account the high fertility rates, early-age pregnancies, and strenuous physical work demanded of women in the study area, our study findings strongly support the argument that prolapse may affect daily life more severely in resource-constrained settings than in more affluent settings [6, 7]. Dynamics of poverty and marginalization in diverse ways reduces the opportunity to receive treatment. The dynamics linked to early marriage and limited schooling among rural Ethiopian women also limits the exchange of knowledge, thus laying the grounds for stigma and discrimination [16, 39]. The majority of the women in this study would not have reached the hospital without being exposed to the mobilization initiative that took place in their communities, providing them with free transport and treatment. Such initiatives are rare in Ethiopia, and they require a long-term sustainable commitment and funding to succeed.

### Strengths and limitations

The topic of prolapse is perceived as extremely sensitive in the current study area, which may have affected the women’s readiness to speak openly. Also, the first author’s sociocultural and language limitations, despite several lengthy research stays in Ethiopia and increasing language competence, are likely to have affected the study’s results. In this context, we would also like to mention that being an outsider can at times be advantageous as one is perceived to be located beyond the locally embedded normative discourse. Moreover, these challenges were partially ameliorated through the follow-up visits to the women at home and the newly gained openness about the condition in the study area. The women also expressed appreciation for the follow-up interviews in their homes after the surgery.

## Conclusions

The present study indicates that the provision of free prolapse treatment in rural Ethiopia has substantial potential in improving the health and social life among affected women. Recruiting women in groups facilitate awareness and empowerment processes that the present study findings suggest may benefit entire communities. Still, supportive systems—not the least economic—may be required to counteract problematic consequences of the surgery, particularly for vulnerable women.

Further research should be conducted on the prevalence and risk factors for prolapse as well as on women’s health-seeking behaviour related to prolapse in resource-constrained settings. Such knowledge is required to inform the development of sustainable interventions for prolapse and other prevalent, treatable, and chronic maternal-health challenges in Ethiopia and similar resource-constrained settings.

## Additional file

Additional file 1: The interview guide used for follow-up interviews with women treated for pelvic organ prolapse. (DOCX 15 kb)

## Abbreviations

HEW: Health-extension worker
NGO: Non-Governmental Organisation
POPQ: Pelvic organ prolapse quantification (staging system)
UNFPA: United Nations Population Fund
