# Supplementary material for: Life after pelvic organ prolapse surgery: a qualitative study in Amhara region, Ethiopia
Source: BMC Womens Health. 2018 May 29;18:74. doi: 10.1186/s12905-018-0568-2 (PMC5975541; doi:10.1186/s12905-018-0568-2)
Supplement: Supplementary file 1 — The interview guide used for follow-up interviews with women treated for pelvic organ prolapse. (DOCX 15 kb) [file 12905_2018_568_MOESM1_ESM.docx]

**Interview guide for women recently treated for pelvic organ prolapse**

1. Can you tell us about how your life-situation has been after the surgery?

Potential probes:

- - Complications after surgery / physical complaints (urinary incontinence? Vault prolapse?)
  - Social implications (disclosure/openness, acceptance from others, husband, intimacy, etc.)
  - Possibility to rest after surgery + abstain from sexual relation

1. Have you been back to hospital for your follow-up appointment?
   - If no: Potential barriers (financial, lack of support from husband, leaving the household and children behind, etc.)
   - If yes: How did you manage to overcome potential barriers?
2. Can you tell us about your experience at the hospital during treatment?
   - How she experienced the surgery and treatment in general
   - Interacting with the staff
   - Interaction with other patients
3. Clarification or follow-up questions based on the informants first interview at the hospital
4. Is there anything else you would like to share with us regarding your condition?
